# 3D free-standing nitrogen-doped reduced graphene oxide aerogel as anode material for sodium ion batteries with enhanced sodium storage

**DOI:** 10.1038/s41598-017-04958-1

**Published:** 2017-07-07

**Authors:** Jiao Zhang, Chuanqi Li, Zhikun Peng, Yushan Liu, Jianmin Zhang, Zhongyi Liu, Dan Li

**Affiliations:** School of Chemistry and Molecular Engineering, Zhengzhou University, 100 Kexue Avenue, Zhengzhou, 450001 P. R. China

## Abstract

Sodium ion batteries have drawn extensive attentions for large-scale energy storage to replace lithium ion batteries primarily due to the natural abundance of sodium resource and low cost, but their energy density and electrochemical performance are hindered by the sluggish diffusion kinetics of sodium ion. Herein, free-standing nitrogen-doped graphene aerogel has been fabricated via hydrothermal reaction as the potential anode material for sodium ion batteries. The three dimensional porous network structure of the graphene aerogel provides sufficient interstitial space for sodium ion accommodation, allowing fast and reversible ion intercalation/de-intercalation. The nitrogen doping could introduce defects on the graphene sheets, making the feasible transport of large-sized sodium ion. Benefiting from the effective structure and nitrogen doping, the obtained material demonstrates high reversible capacities, good cycling performance (287.9 mA h g^−1^ after 200 cycles at a current density of 100 mA g^−1^), especially superior rate capability (151.9 mA h g^−1^ at a high current density of 5 A g^−1^).

## Introduction

Although lithium ion batteries have been regarded as the most competitive choice for high power sources and electric vehicles because of the high energy density, the limited lithium source driven researcher to explore the substitutes for lithium ion batteries^1–3^. Sodium ion batteries have been expected as the most competitive alternative due to their abundance recourse in the earth crust, low price, and similar chemical properties to lithium ion batteries^4–6^. One great challenge in achieving stable cyclability and rate capability for sodium ion batteries is making sure the reversible insertion/extraction of sodium ions, which have larger ionic radius than that of lithium ions^4, 5, 7^. Therefore, it is vital to fabricate a promising electrode material with large surface area to provide more active reaction sites and contact area for electrode materials and electrolyte, high electrical conductivity to ensure fast electron transport, as well as high porosity to access freely sodiation/de-sodiation and shorten the diffusion distance for sodium ions.

Carbonaceous materials, the common anode materials for lithium ion batteries, are considered as the attractive anode candidates for sodium ion batteries because of their good electrical conductivity, environmental friendly, low cost, and stable chemical and physical properties^8^. To date, many carbonaceous material with different structures have been investigated in the electrochemical performance as anode materials for sodium ion batteries, such as carbon fibers^9, 10^, nanowires^11^, carbon nanospheres^12^, reduced graphene oxide^13–15^, and porous carbon composites^16^. Among these materials, graphene is the most attractive anode material for sodium ion batteries due to its extraordinary electric conductivity, high surface area, and remarkable mechanical flexibility. Notably, three dimensional (3D) structured graphene with open channels allows free and fast sodium ion intercalation/de-intercalation. Furthermore, 3D graphene network acted as free-standing anode material can eliminate the loss of electrical contact between the electrode material and current collector, ensuring good electron transport.

In order to improve the specific capacity and energy density, chemical doping, especially nitrogen doping, has attracted great interests in the enhancement of the electrochemical properties of graphene^13, 16, 17^. Firstly, nitrogen doping is believed to increase the electric conductivity by lowering the semiconducting gap^18, 19^. Secondly, the nitrogen doping sites could attract a large number of positive ions due to the higher electronegativity around the doping area^20^, therefore, increase the specific capacity of graphene. Thirdly, nitrogen doping can introduce defects on the graphene sheets^21, 22^. Based on the first-principle’s calculation, the defects can facilitate lithium ion diffusion from the vacancy and go through the graphene sheets due to the lower diffusion barrier around defect sites^21^. These appealing merits driven the study of nitrogen doped graphene as an anode material in the field of sodium ion batteries.

With these motivations, nitrogen-doped graphene (N-doped graphene) aerogel was successfully synthesized via a facile hydrothermal method followed by an annealing treatment using ammonium bicarbonate as nitrogen source. Porous structure with heteroatom doping improves the interface interaction and provides efficient diffusion channels for sodium ions and electrons. The obtained material delivered a reversible capacity of 151.9 mA h g^−1^ at a high current density of 5 A g^−1^ and even maintained a capacity of 287.9 mA h g^−1^ over 200 cycles at a current density of 100 mA g^−1^. The superior electrochemical performance makes the N-doped graphene aerogel material have a promising prospect in practical applications.

## Results and Discussion

**Figure 1:**
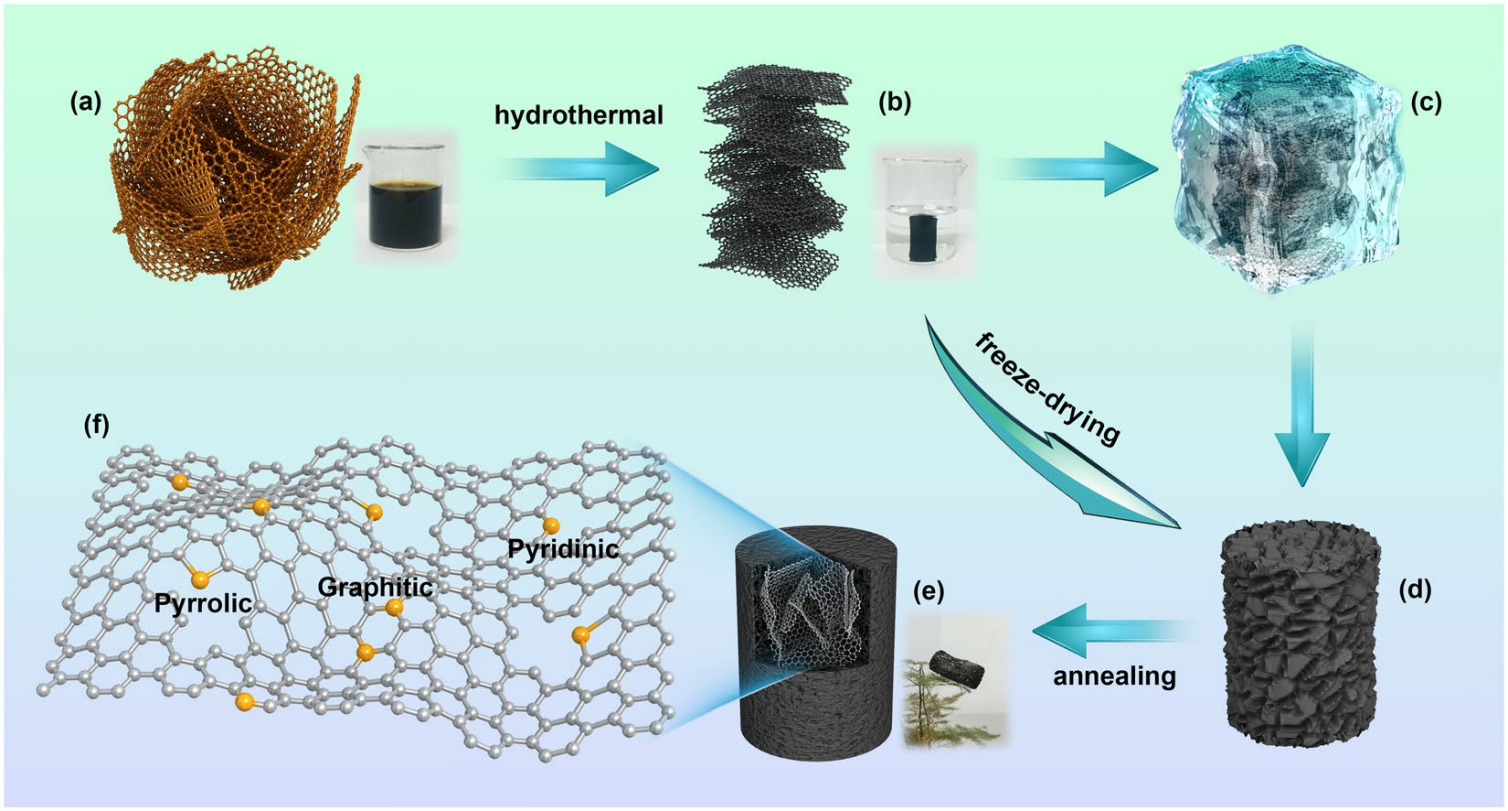
Schematic illustration of synthesis steps for the N-doped graphene aerogel: (**a**) photographic images of graphene oxide suspension (5 mg mL^−1^), (**b**) freeze-dried graphene hydrogel obtained after hydrothermal reaction, (**c**) freeze-drying treatment, (**d**) 3D graphene aerogel, and (**e**) N-doped graphene aerogel. (**f**) Schematic illustration of a N-doped graphene aerogel sheet with three nitrogen doping types.

The synthetic route for the N-doped graphene aerogel is illustrated in Fig. 1. First, GO powder was uniformly dispersed in distilled water by ultrasonication with a concentrate of 5 mg mL^−1^. After a typical hydrothermal reaction of the obtained GO aqueous solution, the reduced graphene oxide sheets assembled to form a black cylindrical hydrogel, which was then freeze-dried to form a 3D porous graphene aerogel. Finally, nitrogen doping in graphene aerogel was conducted by an annealing treatment using ammonium bicarbonate as the nitrogen source. The obtained 3D N-doped graphene aerogel is light and porous, with a low density of 0.034 g cm^−3^ and a good mechanical strength (shown in Fig. S1).

**Figure 2:**
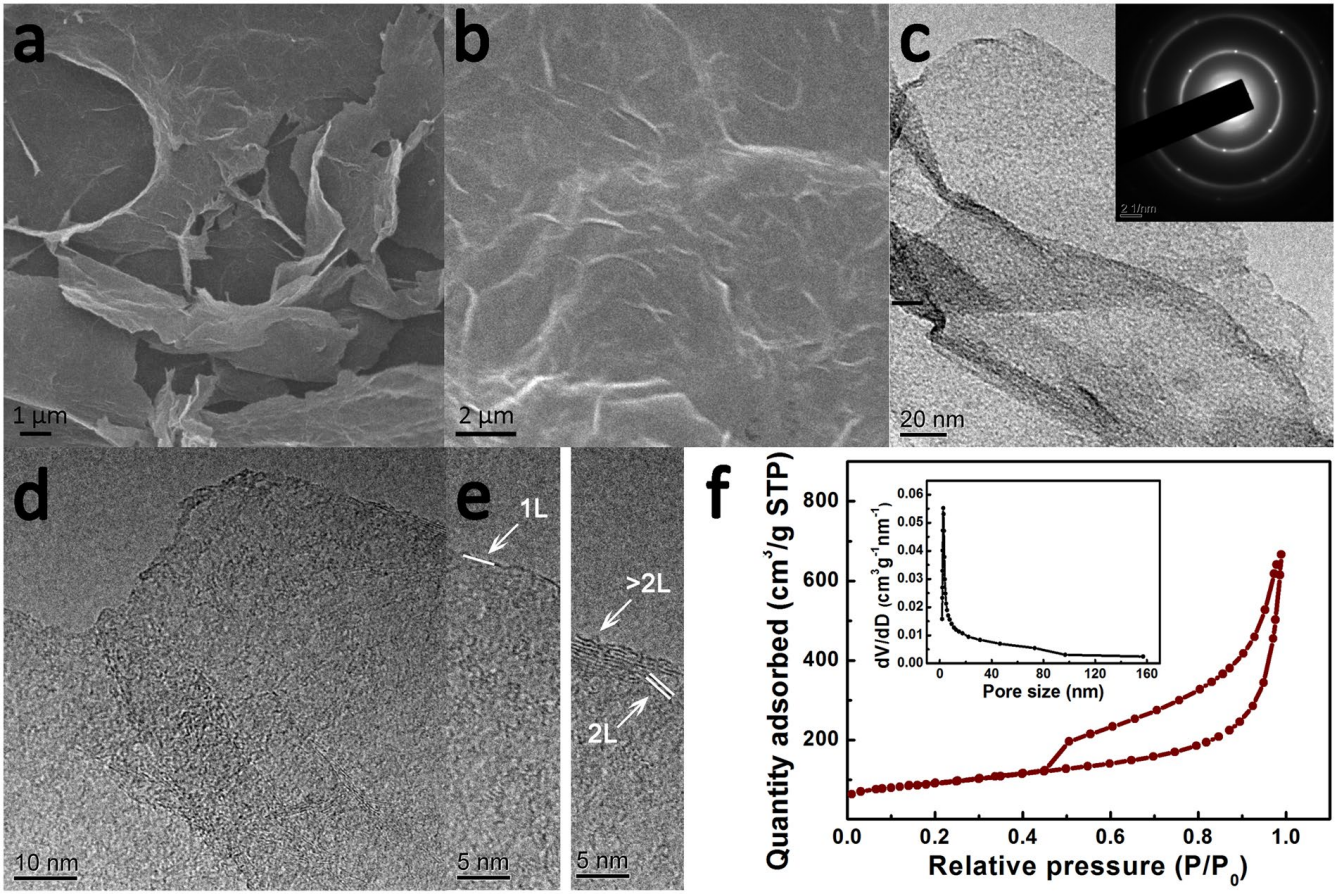
(**a**,**b**) FESEM images of N-doped graphene aerogel; (**c**) TEM image of N-doped graphene aerogel, the inset of (**c**) is the corresponding SAED pattern; (**d**,**e**) HRTEM images of N-doped graphene aerogel; (**f**) Isotherm plot and BJH pore distribution (inset) of N-doped graphene aerogel.

The morphology of the obtained 3D N-doped graphene aerogel after freeze-dried was characterized by filed-emission scanning electron microscopy (FESEM). As shown in Fig. 2a and b, the N-doped graphene nanosheets interconnected, twisted and cross-linked randomly to assemble a 3D framework with opened mesoporous structure. And the pore walls and wrinkles in the surfaces of 3D N-doped graphene aerogel can be seen clearly. More detailed structure was studied by transmission electron microscopy (TEM). It can be observed that the material possesses a thin lamellar structure with distinct edges, overlap and curved profiles (Fig. 2c and d). The inset in Fig. 2c is the corresponding SAED pattern of the N-doped graphene aerogel. More than one set of six-fold symmetric diffraction spots indicate the different orientations of graphene domains^23^. The atomic structure and layers stacking of N-doped graphene aerogel was investigated by High-resolution electron microscopy (HRTEM). Figure 2e confirms the nanosheets in the sample containing both one-, two- and multi-layers. For comparison, the morphology of thermal reduced graphene powder and graphene aerogel was present in Fig. S2. The N_2_ adsorption-desorption curve presents a typical type-IV isotherm and H2 hysteresis loop with a BJH pore distribution ranged from 2.0 to 10.0 nm, which further confirms the mesoporous texture (Fig. 2f). The specific surface areas of N-doped graphene aerogel is 316 m^2^ g^−1^, which was larger than that of pure graphene aerogel (206 m^2^ g^−1^) (Fig. S3).

**Figure 3:**
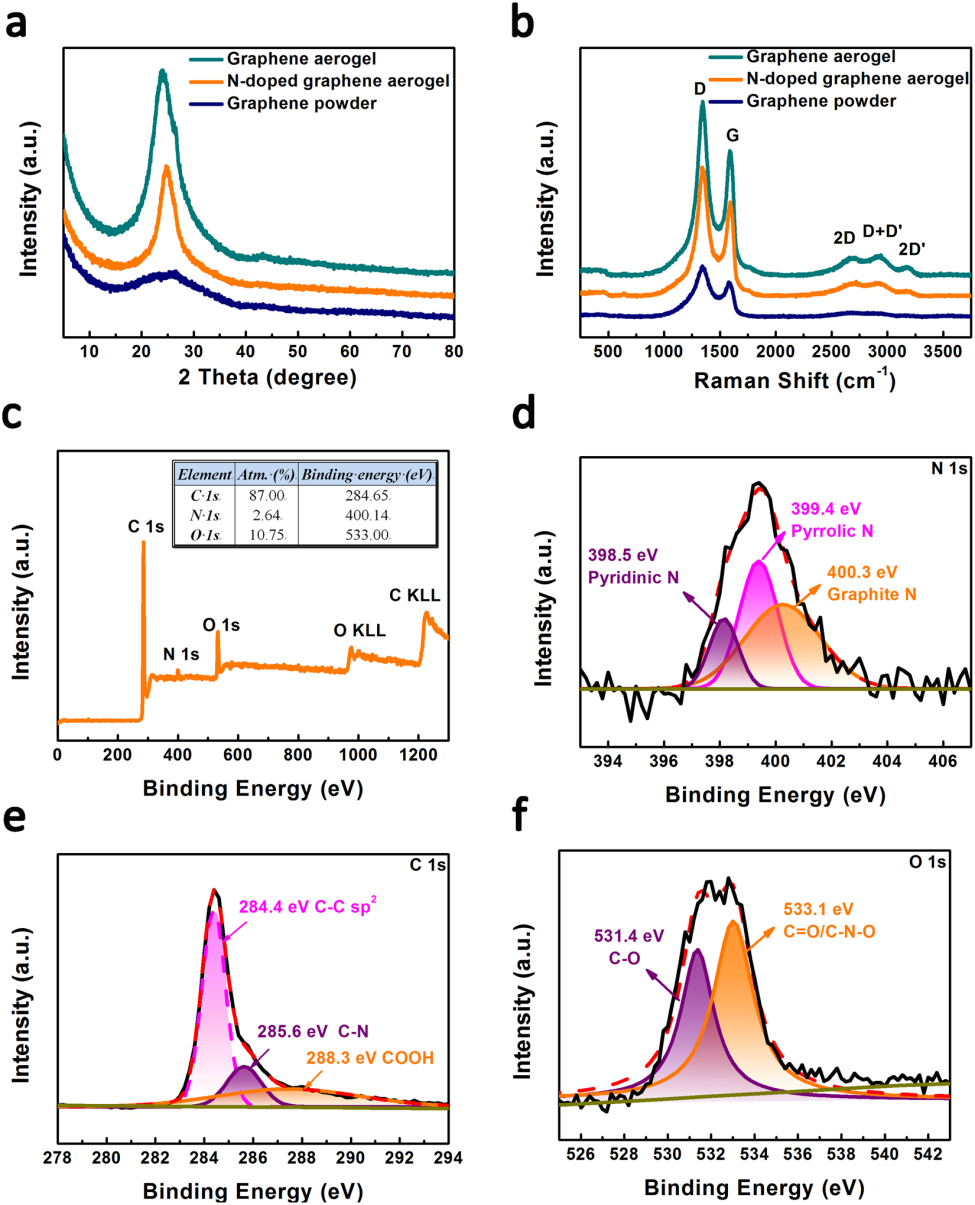
(**a**) Powder X-ray diffraction patterns and (**b**) Raman spectra of the graphene powder, graphene aerogel, and N-doped graphene aerogel samples; (**c**) the XPS survey spectrum of the N-doped graphene aerogel. The inset is the elemental analysis; curve fittings of (**d**) N 1s, (**e**) C 1s and (**f**) O 1s XPS spectra in the N-doped graphene aerogel.

X-Ray diffraction (XRD) patterns of graphene powder, graphene aerogel, and N-doped graphene aerogel are shown in Fig. 3a. After the hydrothermal reaction, the featured diffraction peak of graphene oxide at 10.3° disappeared (Fig. S4), and a new peak near 26° appeared. This indicates the recovery of graphitic crystal structure^24^. The graphene powder shows a broad diffraction peak owning to the stacking of graphene nanosheets. By contrast, the N-doped graphene aerogel exhibits a sharp diffraction peak with high intensity, which may be related to the fine changes of morphology on the surface^25^. Figure 3b shows the Raman spectra of graphene powder, graphene aerogel, and N-doped graphene aerogel samples. Two prominent peaks centered at 1346 cm^−1^ and 1587 cm^−1^ can be observed, which correspond to D band and G band, respectively. The D band is resulted from the defects or structural disorder and G band is accordance with the E_2g_ mode of sp^2^ hybridized carbon atoms^26^. The intensity ratio of the D to G band (I_D_/I_G_) for N-doped graphene aerogel is 1.42, which is higher than that of pristine graphene (1.40) and graphene powder (1.36). This suggests more defects were introduced on the graphene aerogel sheets after nitrogen doping. In addition, the 2D, 2D’ and the combination mode D + D’ are shown in this spectrum of N-doped graphene aerogel, indicating the existence of both monolayer and multilayers of graphene nanosheets^27^.

X-ray photoelectron spectroscopy (XPS) was applied to analyze the element distribution and chemical composition on the surface layer of the N-doped graphene aerogel. Figure 3c exhibits the survey spectrum with main peaks at 284.4, 399.6, and 531.9 eV, corresponding to the C 1s, N 1s, O 1s in the N-doped graphene aerogel, respectively. The result of elemental analysis is shown in the inset. Compared with the survey spectra of graphene powder and graphene aerogel (Fig. S5), the appeared N 1 s peak gives the direct evidence of nitrogen doping in the graphene aerogel sheets. The high resolution spectrum of N 1 s (Fig. 3d) can be resolved into three peaks centered at 398.5 (pyridinic N), 399.4 (pyrrolic N), and 400.2 eV (graphitic N), indicating that nitrogen atoms are incorporated into the carbon–carbon bonds of graphene^28, 29^. The C 1s peak of N-doped graphene aerogel can be fitted into three components at 284.4, 285.6 and 288.3 eV (Fig. 3e), which are ascribed to sp2 hybridized carbon atom, aromatic C-N bonds, and carboxyl group^30^, respectively. The signals of O 1s are attributed to C−O and C=O/C-N-O bonds at 531.4 and 533.1 eV (Fig. 3f)^31^.

**Figure 4:**
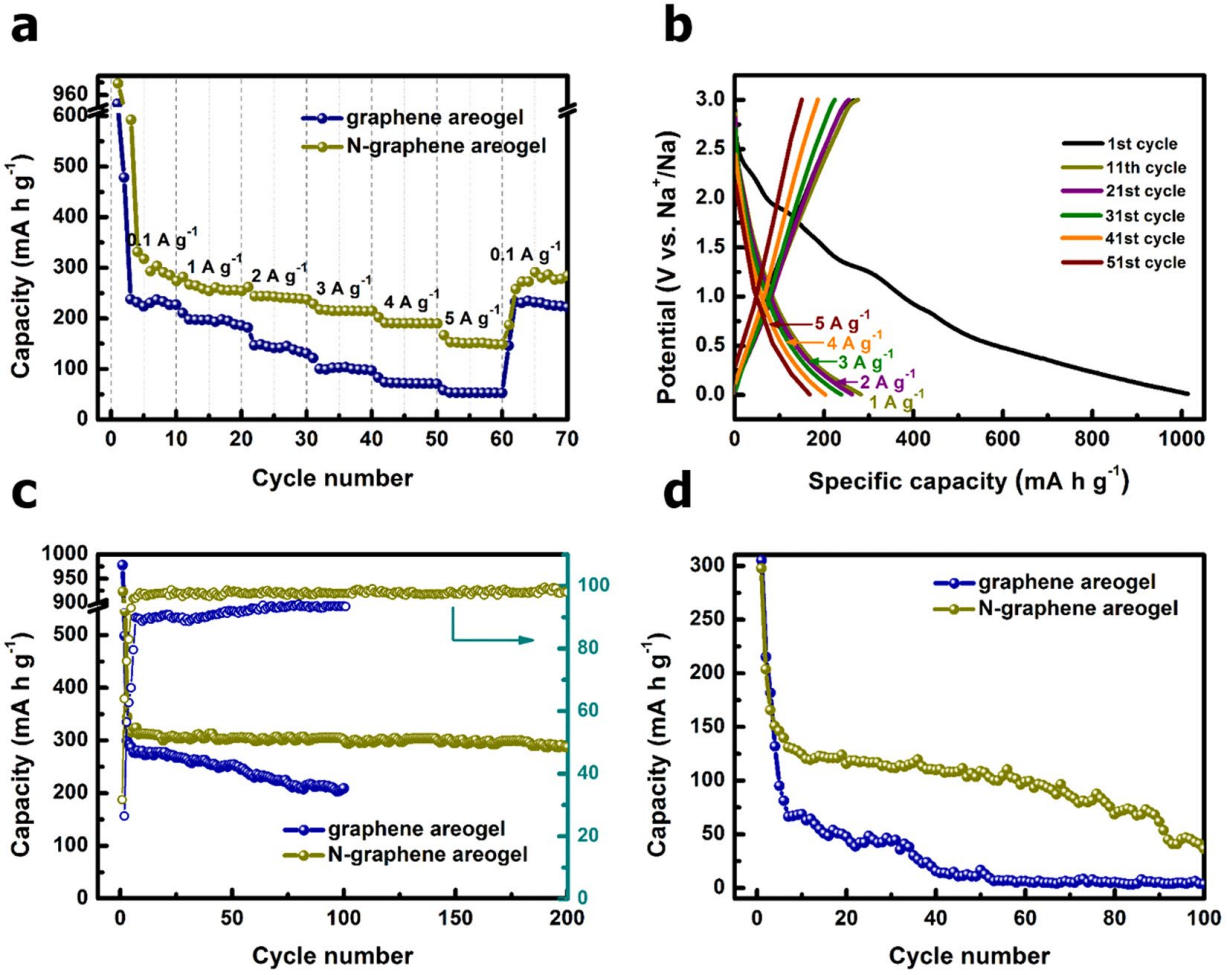
(**a**) Comparison of rate capability of the graphene aerogel and N-graphene aerogel at different current densities from 0.1–5 A g^−1^; (**b**) galvanostatic charge/discharge profiles for the selected cycles of N-graphene aerogel at various current densities from 0.1–5 A g^−1^ (corresponding to (**a**)); comparison of cycling performance of graphene aerogel and N-doped graphene aerogel at a current density of (**c**) 0.1 A g^−1^ and (**d**) 10 A g^−1^. Coulombic efficiencies of the graphene aerogel and N-graphene aerogel were inserted in Fig. 4(c).

The behaviors of sodium ion intercalation/de-intercalation were investigated by a serial of electrochemical tests and shown in Fig. 4. Graphene aerogel was chose as a comparison to gain a better understanding of the effects of nitrogen doping on the electrochemical properties and sodium ion storage mechanism. Figure 4a presents the comparison of rate capability of graphene aerogel and N-doped graphene aerogel at various current densities from 0.1 to 5 A g^−1^. The initial capacity of N-doped graphene aerogel is 1013.8 mA h g^−1^. This high value in capacity is attributed to the electrolyte reductive decomposition, reactions between the oxygen-containing functional groups on the graphene sheets and sodium ions, and the formation of solid electrolyte interphase (SEI) layer^12, 16, 32–35^. The electrode of N-doped graphene aerogel delivered an average reversible capacity of 260.3, 242.9, 215.9, 191.1, and 151.9 mA h g^−1^ at the current densities of 1, 2, 3, 4, and 5 A g^−1^, respectively. It can be observed that there is a steady decrease in capacity from 0.1 to 2 A g^−1^. When the current density came back to 0.1 A g^−1^, the average capacity recovered to 268.3 mA h g^−1^, indicating a good reversibility of the composite. In comparison, the pristine graphene aerogel presents an inferior rate performance, delivering an average of 196.1, 145.5, 102.5, 73.1, and 53.7 mA h g^−1^ at the current density of 1, 2, 3, 4, and 5 A g^−1^, respectively. Figure 4b shows the charge/discharge curves of N-doped graphene aerogel in 1^st^, 11^th^, 21^st^, 31^st^, 41^st^, and 51^st^ cycles at respective current density, which correspond to Fig. 4a. The sloping region could be ascribed to the Na ions storage by defects and/or surface functional groups in the potential range of 0.5–3.0 V, whereas the Na ions intercalation/de-intercalation between interlayers of N-doped graphene aerogel below 0.5 V^36, 37^.

Figure 4c compares the cycling performance of graphene aerogel and N-doped graphene aerogel samples at a constant current density of 0.1 A g^−1^. The N-doped graphene aerogel electrode exhibits an excellent cycling stability over 200 cycles. The capacity underwent a decrease in the first three cycles and became stable from the 4^th^ cycle, which remained a value of 287.9 mA h g^−1^ after 200 cycles with about 98.6% Coulombic efficiency, corresponding to 90.6% of that at the 4^th^ cycle. In terms of the graphene aerogel sample, there is an obvious decaying in capacity during cycling. The specific capacity is 209.2 mA h g^−1^ at the 100^th^ cycle with about 93.4% Coulombic efficiency, which is 71.6% of that at the 4^th^ cycle. The cyclabilities of graphene aerogel and N-doped graphene aerogel were further evaluated a high current density of 5 A g^−1^ for 100 cycles, as shown in Fig. 4d. It can be found that both samples exhibit capacity fading with increasing of cycle number. Compared with N-doped graphene aerogel, the graphene aerogel presents inferior cycling stability, with a capacity dropped dramatically to a value of only 3.4 mA h g^−1^ at the 100^th^ cycle.

The superior electrochemical performance of N-doped graphene aerogel can be attributed to the following essential features. First, the obtained 3D porous framework of N-doped graphene aerogel can provide sufficient interstitial space and more electrochemical active sites for sodium ion reaction and storage, increase the surface area for the contact between active material of electrode and electrolyte, as well as shorten the diffusion distance for sodium ions. Second, the porous structure can accommodate volume change during the sodiation/de-sodiation processes, thus maintain the structural integrity of the electrode material. Third, the robust mechanical flexibility originated from free-standing structure guaranteed an improved cyclability even at high current densities. Forth, the defects introduced by nitrogen doping make the feasible transport of large-sized sodium ion between graphene sheets and also increase the storage sites for sodium ion.

## Conclusions

In summary, 3D free-standing nitrogen-doped graphene aerogel was synthesized by hydrothermal reaction and annealing treatment. The obtained materials exhibited good sodium storage performance, delivering a reversible capacity of 151.9 mA h g^−1^ at a high current density of 5 A g^−1^ and even maintained a capacity of 287.9 mA h g^−1^ after 200 cycles at a current density of 100 mA g^−1^. The superb results demonstrate that the effective 3D framework of N-doped graphene aerogel can provide high porosity with good mechanism properties to tolerate the volume change during charging/discharging processes, increase reaction active sites for sodium ions, and facile sodium ion diffusion. The enhanced electrochemical performance can also be attributed to the nitrogen-doping introduced defects, which is beneficial to the diffusion of large-sized sodium ion during the intercalation/de-intercalation processes. This work proves that the free-standing and binder-free 3D N-doped graphene aerogel is a promising candidate anode material for sodium ion batteries with high energy storage.

## Materials and Methods

### Chemicals

Graphite powder was purchased from Sinopharm Chemical Reagent Co., Ltd. Ammonium bicarbonate (NH_4_HCO_3_), potassium permanganate (KMnO_4_), sodium nitrate (NaNO_3_), hydrogen peroxide (H_2_O_2_), and sulfuric acid (H_2_SO_4_) were analytical grade reagents received from Tianjin Chemical Reagent Technology Co., Ltd. All the chemicals were used without further purification.

### Synthesis of Nitrogen-doped graphene aerogel

Graphene oxide (GO) was synthesized via a modified Hummers method^38^. The three-dimensional (3D) graphene hydrogel was prepared through a hydrothermal process. In a typical experiment, 0.5 g of the obtained GO was dispersed in 100 mL of distilled water under ultrasonication for 4 h and subsequently treated via a hydrothermal reaction at 150 °C for 20 h to form a graphene hydrogel. The hydrogel was freeze-dried to obtain pristine graphene aerogel. To obtain the nitrogen-doped (N-doped) graphene aerogel, the pristine graphene aerogel was loaded in an alumina boat, which was placed in a larger, covered alumina boat containing excess amount of ammonium bicarbonate (6.0 g) outside of the inner alumina boat. Then the alumina boats were transferred to a quartz tube furnace under vacuum and annealed at 300 °C for 2 h. To study the effect of graphene structure on the electrochemical performance of sodium ion batteries, graphene powder without 3D structure was also prepared as a reference from the graphene oxide through a thermal reduction at 800 °C in N_2_ atmosphere with a heating rate of 10 °C min^−1^. The conductivity of N-doped aerogel measured by Four-Point Probe Meter at ambient temperature with a value of 2.4 × 10^−2^ S m^−1^, which is larger than that of graphene aerogel (2.9 × 10^−3^ S m^−1^).

### Electrochemical performance measurements

The as-obtained pristine and N-doped graphene aerogel were pressed under a pressure of 20 MPa and used as the working electrodes without any conductive agent or binder. The average weight of the active material is about 0.60 g cm^−2^ in the electrode. The electrochemical tests were carried out with CR2032 coin type cells. The cells were constructed with sodium foil as the anode, the prepared active material as the cathode, glass microfiber as the separator, and 1 M NaClO_4_ in a mixture of ethylene carbonate (EC) and propylene carbonate (PC) (1:1 by volume) as the electrolyte (about 4 drops). The whole assembly process was carried out in an argon-filled glove box. The charge-discharge cycling was performed within the voltage range of 0.01–3 V vs. Na^+^/Na on a battery test instrument (LAND CT2001A, KINGNUO, China) at ambient temperature. Cyclic voltammetry (CV) and electrochemical impedance spectroscopy (EIS) measurements of the electrodes were carried out on an electrochemical workstation (CHI660E). The cyclic voltammograms were obtained over the potential range from 0.01 to 3.0 V at varous scan rate from 0.1 to 5 mV s^−1^. The EIS was conducted by applying a dc potential equal to open circuit voltage over the frequency range from 100 kHz to 0.01 Hz with an amplitude of 5 mV.

### Characterization

The morphologies and microstructures of the resulting materials were characterized by transmission electron microscope (TEM, JEM-2100F, JEOL, 200 kV) and field-emission scanning electron microscope (FESEM, JSM-7500F, JEOL, 5 kV). X-ray diffraction analysis were performed on a PANalytica X’PERT PRO with Cu Kα (λ = 1.541 Å) over the range from 2*θ* = 10° to 90°. Raman spectra were recorded on a LabRAM HR Evolution with the laser excitation of 532 nm. X-ray photoelectron spectroscopy (XPS) experiments were conducted on an ESCALAB 250Xi X-ray photoelectron spectrometer. The electrical conductivities were measured using SZT-2A Four-Point Probe Meter at ambient temperature. Brunauer−Emmett−Teller (BET) measurement was carried out on a Quantachrome NOVA 1000e instrument by N_2_ physisorption at 77 K. Macropores distribution was analyzed on AutoPore IV 9500 instrument (Micromeritics).

## Electronic supplementary material

Supplementary Information
