# Supplementary material for: 3D free-standing nitrogen-doped reduced graphene oxide aerogel as anode material for sodium ion batteries with enhanced sodium storage
Source: Sci Rep. 2017 Jul 7;7:4886. doi: 10.1038/s41598-017-04958-1 (PMC5501838; doi:10.1038/s41598-017-04958-1)
Supplement: Supplementary file 1 — Supplementary Information [file 41598_2017_4958_MOESM1_ESM.doc]

**Supporting Information**

**3D free-standing nitrogen-doped reduced graphene oxide aerogel as anode material for sodium ion batteries with enhanced sodium storage**

Jiao Zhang, Chuanqi Li, Zhikun Peng, Yushan Liu, Jianmin Zhang, Zhongyi Liu* and Dan Li*

College of Chemistry and Molecular Engineering, Zhengzhou University, 100 Kexue Avenue, Zhengzhou 450001, P R China

* Corresponding Authors. Email: liuzhongyi@zzu.edu.cn and danli@zzu.edu.cn


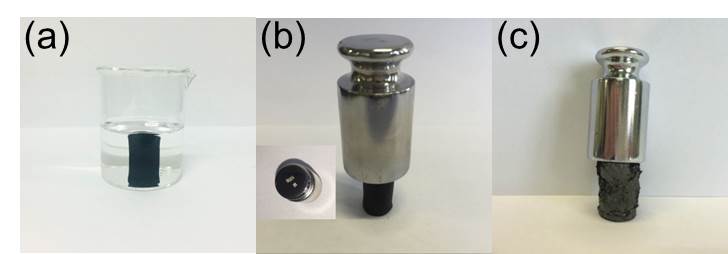


**Fig. S1** (a) Photograph of the obtained pristine graphene hydrogel by a hydrothermal reduction of 5 mg mL^-1^ of GO solution at 150 ºC for 20 h. (b) Mechanical compression test of pristine graphene hydrogel by supporting the weight of 100 g. (c) Mechanical compression test of N-doped graphene aerogel by supporting the weight of 100 g.


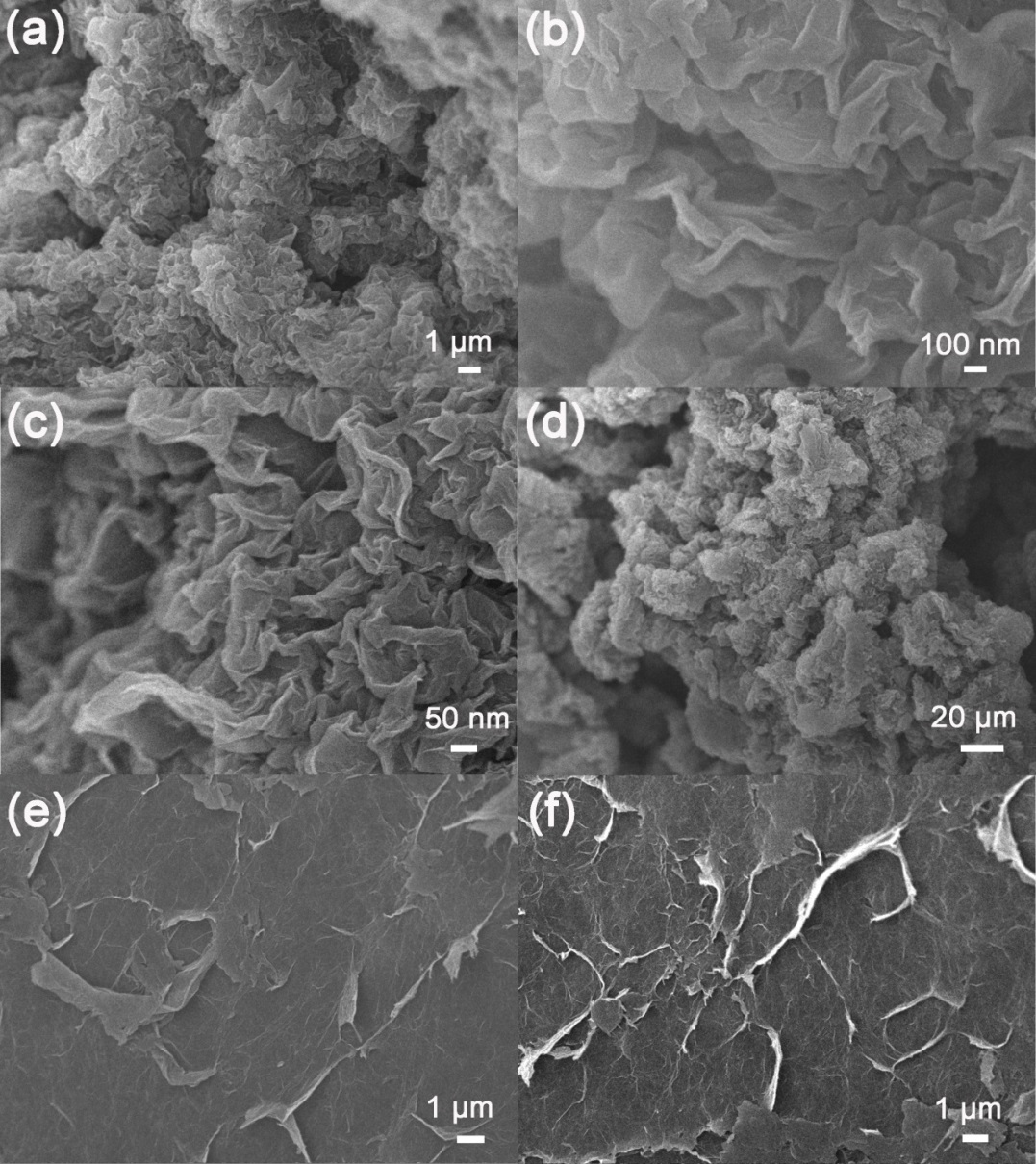


**Fig. S2** SEM images of graphene powder (a-d) and graphene aerogel (e, f).


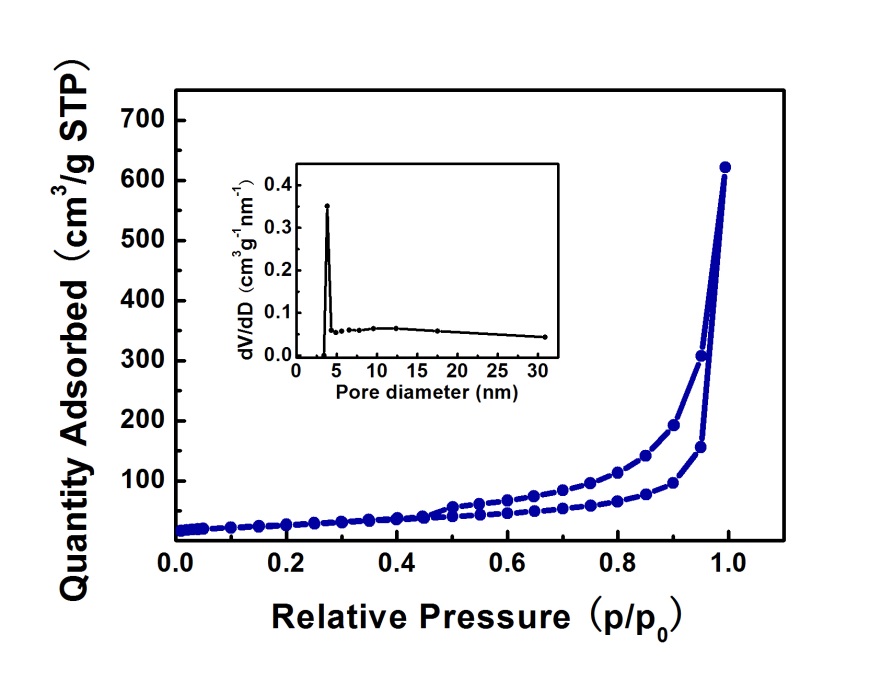


**Fig. S3** Isotherm plot and BJH pore distribution of graphene aerogel.


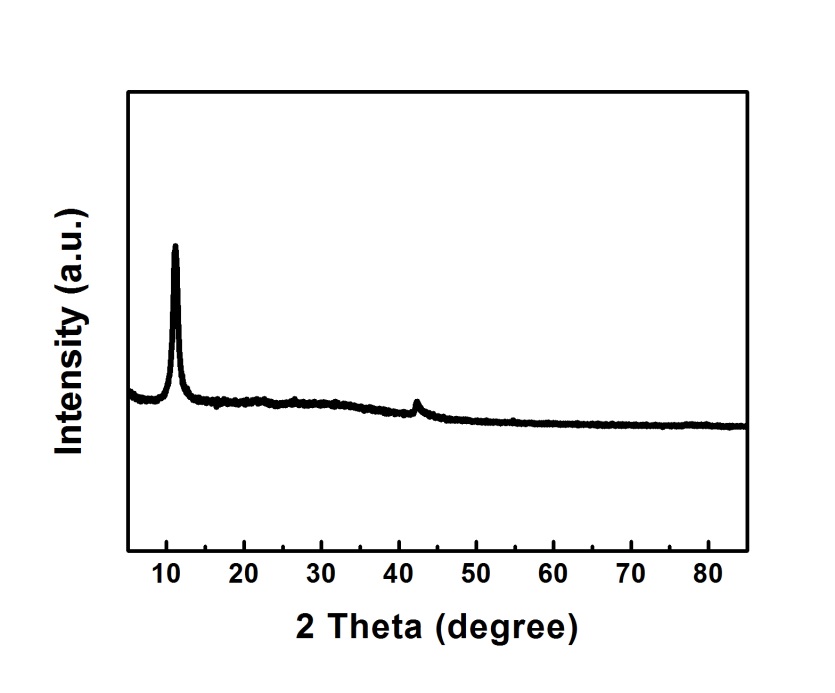


**Fig. S4** XRD pattern of graphene oxide.


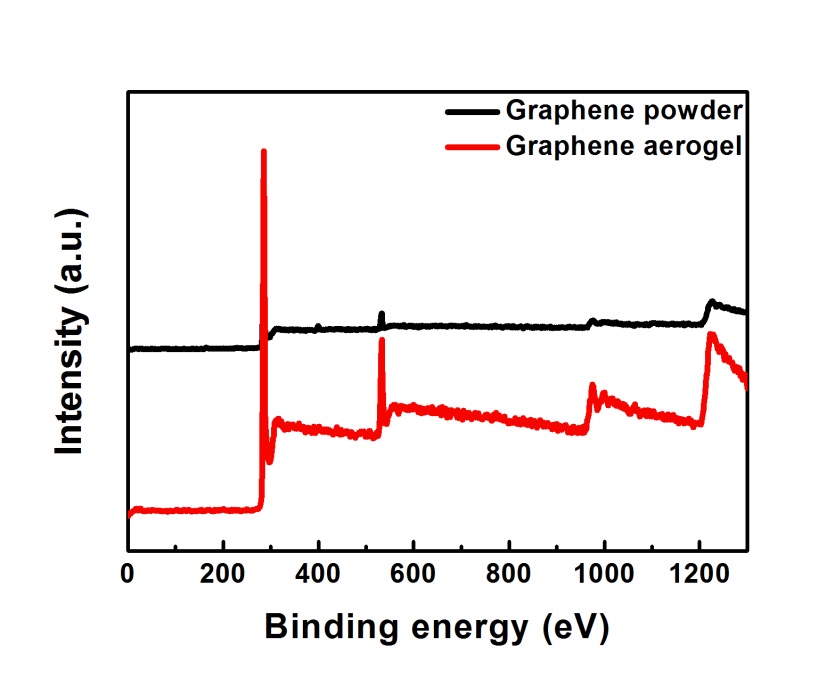


**Fig. S5** the survey XPS spectra of graphene powder and graphene aerogel.


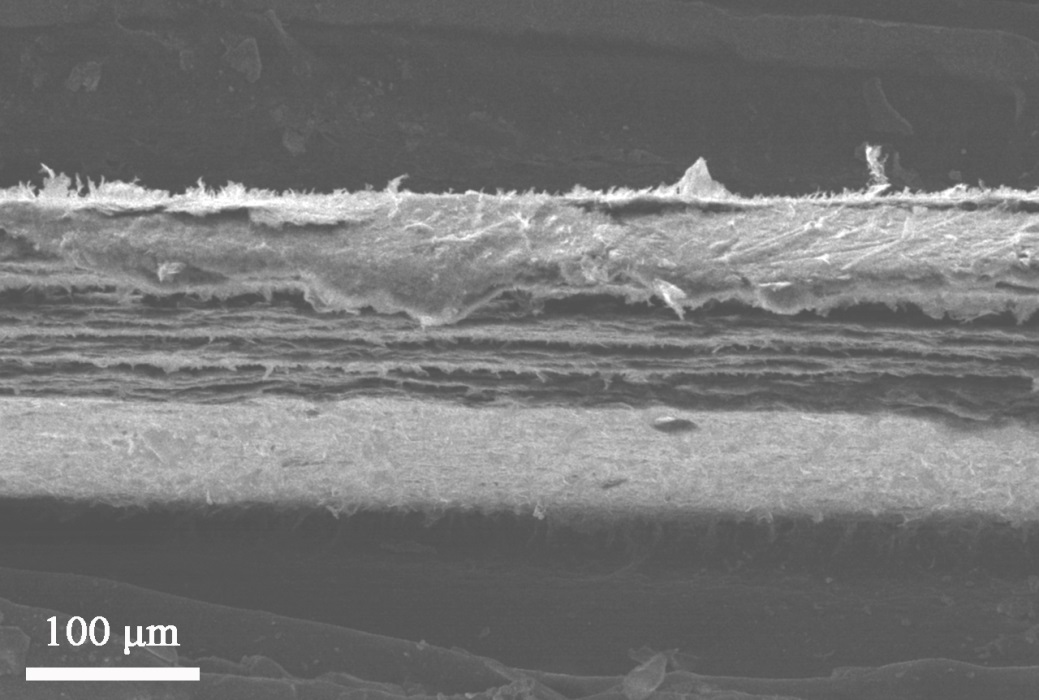


**Fig. S6** SEM images of the cross section of N-doped graphene aerogel electrode after pressing under 20 MPa.

The thickness of (N-doped) graphene electrode after pressing was about 0.18 mm.


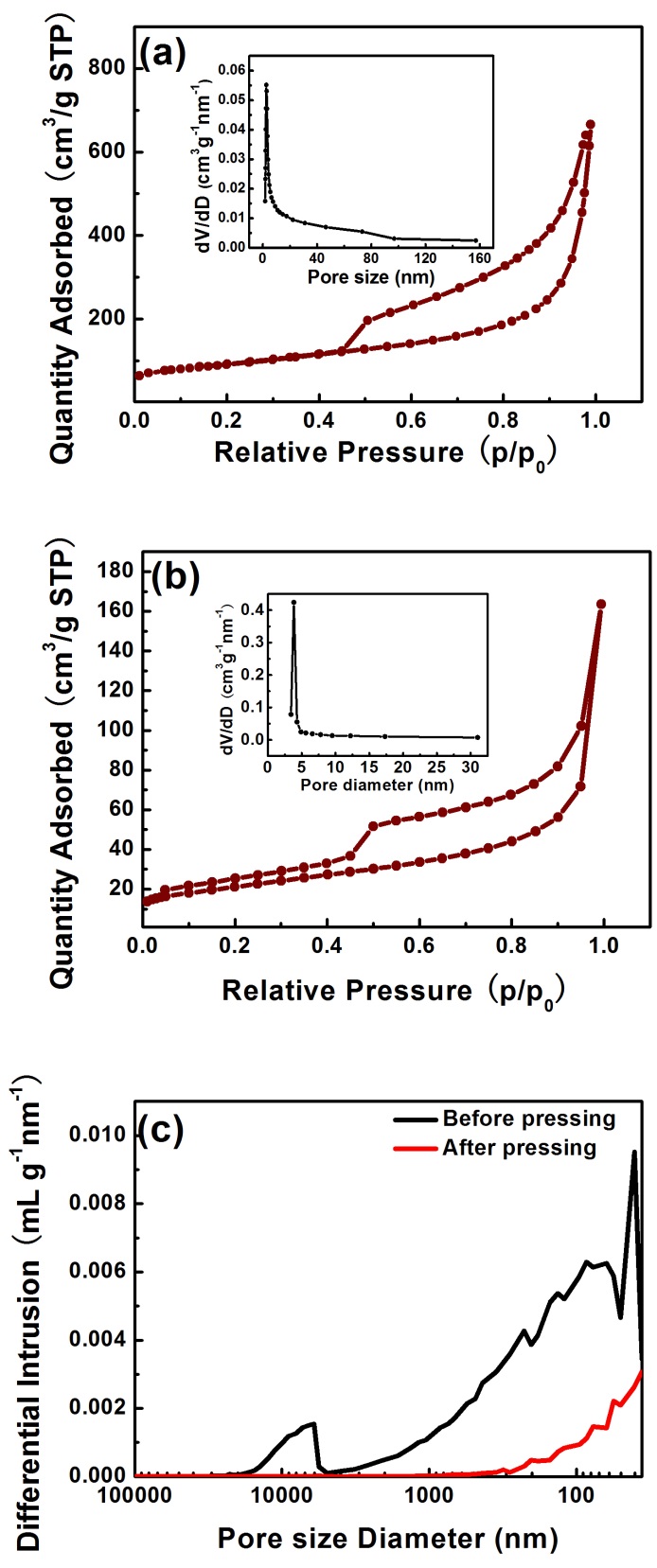


**Fig. S7** N_2_ adsorption-desorption isotherms of N-doped graphene aerogel before (a) and after pressing (b) under 20 MPa, inset is the corresponding BJH pore size distribution curves. (c) Macropores distribution curves of N-doped graphene aerogel before and after pressing under 20 MPa measured by Mercury intrusion porosimetry.

**Table S1.** Microstructure parameters and composition of the carbons.

| Samples | N-doped graphene aerogel | | Graphene aerogel |
| --- | --- | --- | --- |
|  | Before pressing | After pressing |  |
| S_BET_ (m^2^ g^-1^) | 316 | 92 | 206 |
| V (cm^3^ g^-1^) | 1.03 | 0.15 | 0.51 |


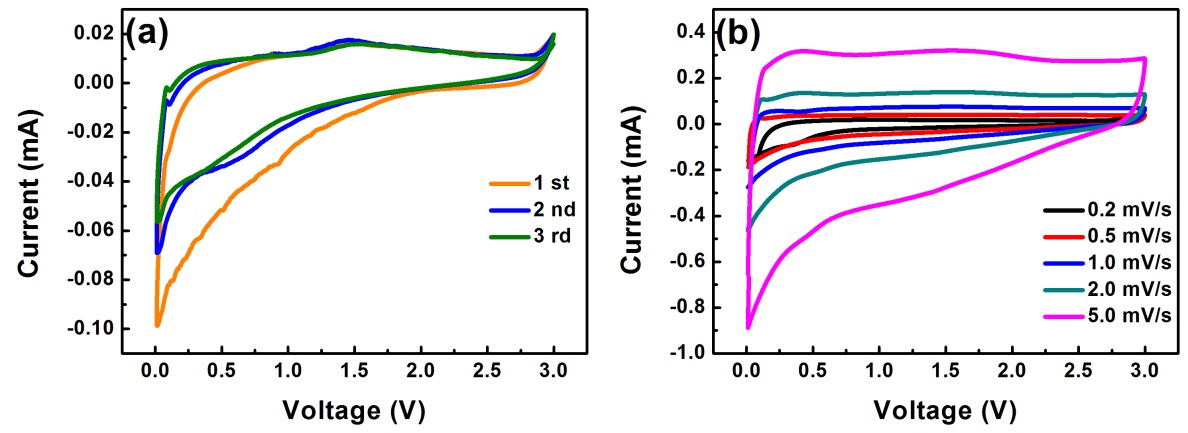


**Fig. S8** Cyclic voltammetry of N-doped graphene aerogel at the scan rate of (a) 0.1 mV s^-1^ and (b) 0.2-5.0 mV s^-1^ between 0.01 and 3.0 V.


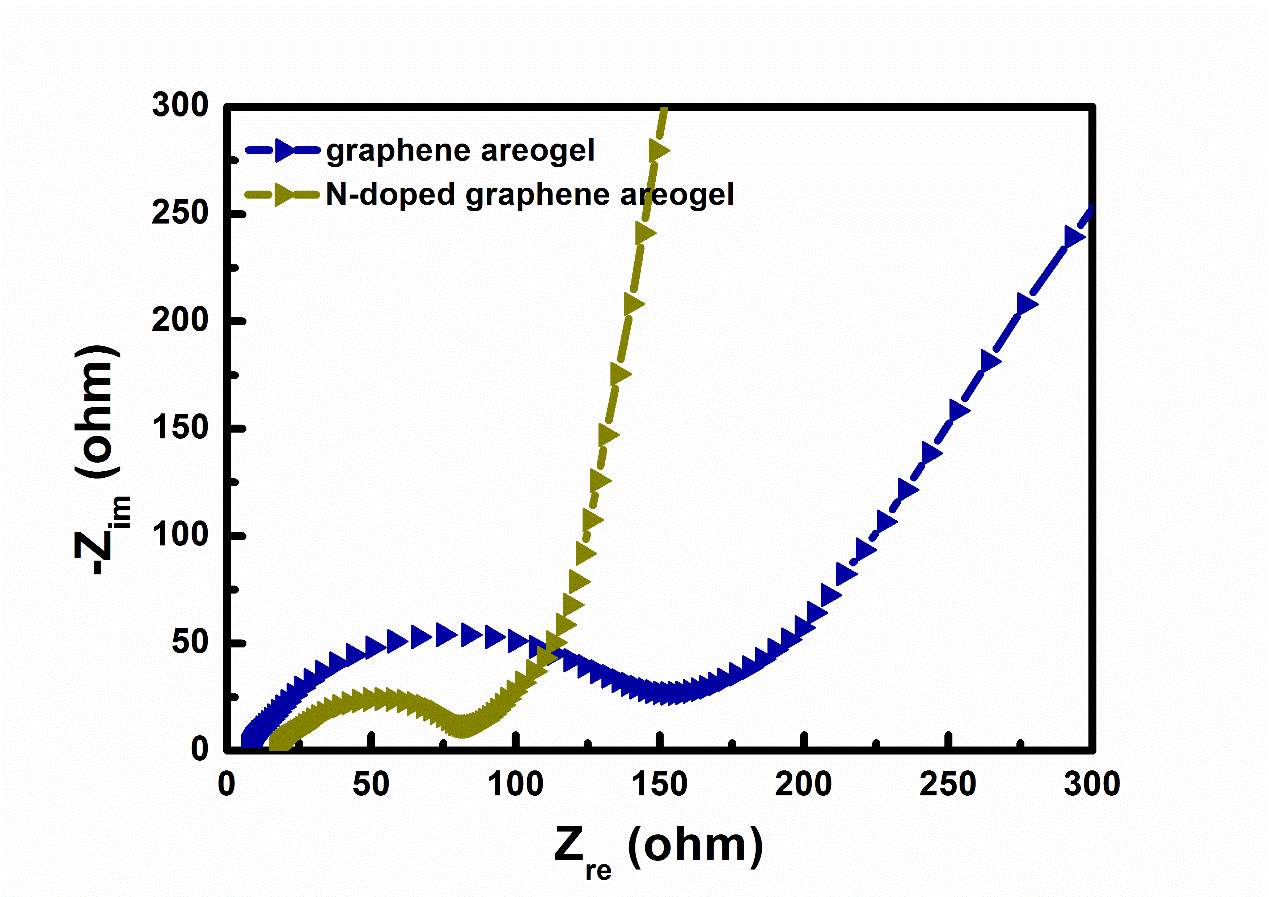


**Fig. S9** Electrochemical impedance spectra of graphene aerogel and N-doped graphene aerogel.

**Table S2.** Comparison of the electrochemical performance of graphene-based aerogel materials.

| **Strategies** | **Materials** | **Cycling performance**  **(mA h g^-1^)** | **Rate Capability**  **(mA h g^-1^)** | **Ref.** |
| --- | --- | --- | --- | --- |
| Li-ion battery | N-Doped Graphene Sheets | 872 after 30 cycles | 1040 at 50 mA g^-1^ | [1] |
|  | N-Doped Holey Graphene | 800 mA cm^-3^ after 1200 cycles at 3.2 mA cm^-2^ | 1100 mA cm^-3^ at 0.1 mA cm^-2^ | [2] |
|  | Reduced Graphene Oxide | 346 after 60 cycles | 1580 at 0.5C | [3] |
|  | Nitrogen-Doped Graphene Films | 0.06 mA h cm^-2^ after 50 cycles | 0.09 mA h cm^-2^ at  1 μA cm^-2^ | [4] |
|  | N-doped Graphene and Graphite Composites | 98.1% after 1000 cycles | 781 at 0.1C | [5] |
|  | Photothermally Reduced Graphene | 156 over 1000 cycles | 375 at 5C | [6] |
|  |  |  |  |  |
| Na-ion battery | Reduced Graphene Oxide/Carbon  Nanotube Sponge | 195 after 7440 cycles | 560 at 50 mA g^-1^ | [7] |
|  | Nitrogen-Doped Carbon/Graphene Hybrid | 280 after 200 cycles | 336 at 30 mA g^-1^ | [8] |
|  | Nitrogen Doped Graphene Nanosheets | 90 after 600 cycles | 260 at 50 mA g^-1^ | [9] |
|  | S-Doped N-Rich Carbon Nanosheets with Expanded Interlayer | 221 after 1000 cycles | 350 at 50 mA g^-1^ | [10] |
|  | Reduced Graphene Oxide | 200 after 750 cycles at 1.0 A g^-1^ | 450 at 25 mA g^-1^ | [11] |
|  | N-doped aerogel | 287.9 after 200 cycles at 100 mA g^-1^ | 1013.8 at 100 mA g^-1^ | This work |

**References**

[1] Wu, Z., Ren, W., Xu, L., & Li, F. Doped graphene sheets as anode materials with superhigh rate and large capacity for lithium ion batteries. *ACS Nano* **5**, 5463-5471 (2011).

[2] Wang, X. et al. High-density monolith of N-doped holey graphene for ultrahigh volumetric capacity of Li-ion batteries. *Adv. Energy Mater.* **6**, 1502100 (2016).

[3] Li, J. et al. Synthesis of few-layer reduced graphene oxide for lithium-ion battery electrode materials. *Ind. Eng. Chem. Res.* **53**, 13348-13355 (2014)

[4] Reddy, A. L. M. et al. Synthesis of Nitrogen-doped graphene films for lithium battery application. *ACS Nano* **4**, 6337-6342 (2010).

[5] Wu, G. et al. N-doped graphene/graphite composite as a conductive agent-free anode material for lithium ion batteries with greatly enhanced electrochemical performance. *Electrochim. Acta* **171**, 156–164 (2015).

[6] Mukherjee, R., Thomas, A. V., Krishnamurthy, A., & Koratkar, N. Photothermally reduced graphene as high-power anodes for lithium-ion batteries. *ACS Nano* **6**, 7867-7878 (2012).

[7] Yan, D. et al. Reduced graphene oxide/carbon nanotubes sponge: A new high capacity and long life anode material for sodium-ion batteries. *J. Power Sources* **316**, 132-138 (2016).

[8] Liu, H. et al. Nitrogen-doped carbon/graphene hybrid anode material for sodium-ion batteries with excellent rate capability. *J. Power Sources* **319**, 195-201 (2016).

[9] Ma, G., Huang, K., Zhuang, Q., & Ju, Z. Superior cycle stability of nitrogen-doped graphene nanosheets for Na-ion batteries. *Mater. Lett.* **174**, 221-225 (2016).

[10] Yang, J. et al. S-doped N-rich carbon nanosheets with expanded interlayer distance as anode materials for sodium-ion batteries. *Adv. Mater.* **29**, 1604108 (2017).

[11] Wan, J. et al. In situ transmission electron microscopy observation of sodiation-desodiation in a long cycle, high-capacity reduced graphene oxide sodium-ion battery anode. *Chem. Mater.* **28**, 6528-6535 (2016).
